# Associations of Plasma and CSF Osteocalcin Levels With CSF ATN Biomarkers and Cognitive Functions in Alzheimer's Disease

**DOI:** 10.1002/mco2.70255

**Published:** 2025-06-19

**Authors:** Xian‐Le Bu, Zhuo‐Ting Liu, Jia‐Yan Xin, Mei Huang, Yu‐Di Bai, Jin Zhou, Yun‐Yu Bao, Jiang‐Hui Li, Zhi‐Hao Liu, Gui‐Hua Zeng, An‐Yu Shi, Dong‐Wan Chen, Yu‐Jie Lai, Yang Chen, Fan Zeng, Jun Wang, Qing‐Qing Tao, Zhi‐Ying Wu, Yan‐Jiang Wang

**Affiliations:** ^1^ Department of Neurology and Centre for Clinical Neuroscience Daping Hospital Third Military Medical University Chongqing China; ^2^ Chongqing Key Laboratory of Ageing and Brain Diseases Chongqing China; ^3^ Institute of Brain and Intelligence Third Military Medical University Chongqing China; ^4^ State Key Laboratory of Trauma and Chemical Poisoning (Third Military Medical University) Chongqing China; ^5^ Key Laboratory of Geriatric Cardiovascular and Cerebrovascular Disease Research Ministry of Education of China Chongqing China; ^6^ Department of Medical Genetics and Center for Rare Diseases and Department of Neurology in Second Affiliated Hospital and Key Laboratory of Medical Neurobiology of Zhejiang Province Zhejiang University School of Medicine Hangzhou Zhejiang China

**Keywords:** alzheimer's disease, amyloid‐beta, cognitive function, osteocalcin, tau

## Abstract

Animal studies have shown that osteocalcin (OCN), a hormone derived from bone, plays a vital role in brain development and cognitive function. However, its potential connection to Alzheimer's disease (AD) pathology in humans remains largely unexplored. This cross‐section study included 238 cognitively unimpaired participants, 26 mild cognitive impairment (MCI) patients, 54 AD dementia patients, and 32 patients with non‐AD neurodegenerative diseases. Plasma and cerebrospinal fluid (CSF) levels of OCN were measured by enzyme‐linked immunosorbent assay kits. In the clinical diagnosis‐based subgroup, plasma and CSF levels of OCN were significantly higher in MCI and AD dementia compared with cognitively unimpaired participants. In the ATN framework‐based subgroup, plasma and CSF OCN levels were significantly elevated in Aβ‐positive participants, including those in the preclinical stage of AD. Both plasma and CSF OCN levels were negatively correlated with CSF Aβ42 and positively correlated with CSF total‐tau and phosphorylated‐tau181/Aβ42. In addition, OCN mediated the relationship between Aβ pathology and tau pathology. Notably, OCN levels in plasm and CSF were also negatively associated with cognitive functions. This study provides clinical evidence linking OCN to AD, suggesting that OCN may be associated with brain Aβ deposition, tau hyperphosphorylation and neurodegeneration.

## Graphical abstract

In this study, we observed elevated levels of plasma and cerebrospinal fluid (CSF) osteocalcin (OCN) in patients with preclinical Alzheimer's disease (AD), mild cognitive impairment, and AD dementia. Both plasma and CSF OCN levels were correlated with brain amyloid‐β (Aβ) deposition, tau hyperphosphorylation, neurodegeneration, and cognitive functions. Additionally, CSF OCN mediated the relationship between Aβ pathology and tau pathology. This study provides clinical evidence linking OCN to AD, suggesting that OCN may play a role in the pathogenesis of AD.

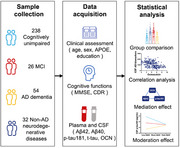

## 1 Introduction

Alzheimer's disease (AD) is the most common neurodegenerative disease and the most prevalent type of dementia [1]. Clinically, it is marked by cognitive decline and behavioral impairments, while its key pathological features include the extracellular amyloid‐β (Aβ) plaques and twisted tau protein strands (tangles) inside brain neurons [2]. AD is a multifactorial condition influenced by a combination of factors, including age, sex, genetics, metabolism, inflammation, and environmental exposures [3]. Despite extensive research, the underlying mechanisms of AD pathogenesis remain poorly understood.

Traditionally, AD has been viewed as a disorder confined to the brain. However, growing evidence suggests that systemic disorders and peripheral factors play a significant role in its onset and progression [4]. Therefore, AD is increasingly regarded as a systemic condition, implying that its onset and progression may involve a complex interplay of processes interacting with peripheral systems [5, 6, 7].

Bone, the structural foundation of the human body, also serves an important endocrine function. By releasing bone‐derived signaling molecules, it influences the activity and health of organs beyond the skeletal system [8]. Recent studies have shown that several bone‐derived molecules, including osteocalcin (OCN), play a role in brain function and neurological disorders [8, 9, 10]. Among these molecules, OCN, the most abundant noncollagenous protein produced by bone, can cross the blood–brain barrier (BBB) and bind to receptors in specific brain regions [11, 12, 13]. Emerging evidence highlights OCN's involvement in various processes, including brain development [11], parkinsonian neurodegeneration [14, 15], neurotransmitter synthesis [11], oligodendrocytes myelination [13], and cognition [12, 16]. Additionally, elevated blood OCN levels have been observed in patients with osteoporosis, a known risk factor for AD [17, 18, 19]. These findings suggest a potential link between OCN and AD.

Therefore, the present study aimed to investigate changes in OCN levels in blood and cerebrospinal fluids (CSF) among cognitively unimpaired (CU) participants, patients with mild cognitive impairment (MCI) or AD dementia. Furthermore, we explored the associations between OCN levels and cognitive functions as well as CSF core AD biomarkers. We found that plasma and CSF levels of OCN were significantly higher in patients with preclinical AD, MCI, and AD dementia compared with CU participants. Plasma and CSF OCN levels were correlated with cognitive functions and CSF AD biomarkers. In addition, CSF OCN mediated the relationship between Aβ pathology and tau pathology. Overall, this study provides clinical evidence for the relationship between OCN and AD, suggesting that OCN may be a key bone‐derived factor mediating the involvement of the bone–brain axis in AD.

## 2 Results

### 2.1 Demographic and Clinical Characteristics

The characteristics of the participants from Chongqing Ageing & Dementia Study (CADS) were summarized in Table 1. The preclinical AD group had a higher age than the CU‐non AD, suspected non‐AD pathophysiology (SNAP) and AD dementia groups. The AD dementia group included a higher proportion of females compared with the CU‐non AD and preclinical AD groups. Additionally, participants in the AD dementia group had lower levels of education than those in the CU‐non AD and MCI groups. The percentage of APOE ε4 carriers was higher in the MCI and AD dementia groups compared with the CU‐non AD and SNAP groups. As expected, patients with MCI and AD dementia scored lower on Mini–Mental State Examination (MMSE) and higher on clinical dementia rating (CDR) scale compared with the CU groups. The AD dementia group also performed worse on MMSE and CDR compared with the MCI group. No significant differences were observed in the prevalence of diabetes and hypertension across groups. However, except for MMSE and CDR, the effect sizes for age, sex, education, APOE genotype, diabetes, and hypertension were small to moderate. Patients with MCI and AD dementia exhibited lower CSF Aβ42 levels and higher total tau (t‐tau) and phosphorylated tau (p‐tau)181 levels compared with the CU groups.

**TABLE 1:** Demographics and clinical data of the participants in the Chongqing cohort.

	Cognitively unimpaired (CU)						
Variable	CU‐non AD (A−TN−, *n* = 136)	Preclinical AD (A+, *n* = 58)	SNAP (A−TN+, *n* = 44)	MCI (*n* = 26)	AD dementia (*n* = 54)	*p* Values	*η* ^2^/Cramer's *V*
Age, mean (SD), y	61.5 (13.6)	70.5 (12.9) ^a^	62.2 (10.6)^b^	68.3 (7.9)	63.4 (9.2)^b^	<0.001	0.080
Female, *n* (%)	50 (36.8)	13 (22.4)	17 (38.6)	11 (42.3)	32 (59.3)^a^, ^b^	0.002	0.228
Education, mean (SD), y	11.1 (2.3)	9.9 (2.6)	10.8 (2.2)	12.2 (3.1)^b^	9.6 (2.6)^a^, ^b^	<0.001	0.096
APOE ε4 carriers, *n* (%)	17 (12.5)	19 (32.8)^a^	8 (18.2)	11 (42.3)^a^, ^c^	26 (48.1)^a^, ^c^	<0.001	0.324
MMSE, mean (SD)	27.4 (2.2)	26.5 (2.9)	27.2 (1.9)	22.4 (5.4)^a^, ^b^, ^c^	12.3 (5.5)^a^, ^b^, ^c^, ^d^	<0.001	0.745
CDR, mean (SD)	0	0	0	0.5 (0)^a^, ^b^, ^c^	1.6 (0.7)^a^, ^b^, ^c^, ^d^	<0.001	0.811
Diabetes, *n* (%)	12 (8.8)	8 (13.8)	5 (11.4)	4 (15.4)	3 (5.4)	0.492	0.103
Hypertension, *n* (%)	33 (24.3)	25 (43.1)^a^	7 (15.9)^b^	7 (26.9)	7 (13.0)^b^	0.003	0.227
CSF biomarkers							
Aβ42, mean (SD), pg/mL	1438.3 (285.2)	704.4 (160.1)^a^	1646.0 (281.8)^a^, ^b^	733.25 (234.0)^a^, ^c^	580.2 (221.2)^a^, ^c^	<0.001	0.723
Aβ40, mean (SD), pg/mL	10944.3 (3390.6)	7462.2 (4339.0)^a^	14174.3 (4218.0)^a^, ^b^	10515.6 (3362.1)^b^, ^c^	10016.3 (4866.3)^b^, ^c^	<0.001	0.207
T‐tau, mean (SD), pg/mL	147.9 (54.3)	232.8 (117.1)^a^	269.1 (98.8)^a^	302.7 (230.2)^a^	388.7 (229.0)^a^, ^b^, ^c^	<0.001	0.302
P‐tau181, mean (SD), pg/ mL	35.6 (8.2)	44.7 (18.7)	61.6 (14.9)^a^, ^b^	54.5 (45.6)^a^ ^,^	72.0 (45.3)^a^, ^b^, ^d^	<0.001	0.240

*Note*: Categorical variables are presented as numbers and percentages; continuous variables are presented as mean ± SD. The *p*‐value in the penultimate column indicates the statistical significance of group comparisons conducted using one‐way analysis of variance and chi‐square test. Effect sizes for ANOVA and chi‐square tests were quantified using *η* ^2^ and Cramer's *V*, respectively.

Abbreviations: A+, Aβ‐positive; AD, Alzheimer's disease; ANOVA, analysis of variance; APOE, apolipoprotein E; A−TN−, negative Aβ, p‐tau and t‐tau; A−TN+, Aβ‐negative and p‐tau or t‐tau‐positive; Aβ40, amyloid‐β 1–40; Aβ42, amyloid‐β 1–42; CDR, clinical dementia rating; CSF, cerebrospinal fluid; CU, cognitive unimpaired; MCI, mild cognitive impairment; MMSE, Mini–Mental State Examination; *n*, number; P‐tau181, phosphorylated tau 181; SD, standard deviation; SNAP, suspected non‐AD pathophysiology; T‐tau, total tau; y, years.

^a^ *p* < 0.05 compared with CU‐non AD.

^b^ *p* < 0.05 compared with preclinical AD.

^c^ *p* < 0.05 compared with CU‐SNAP.

^d^ *p* < 0.05 compared with MCI.

### 2.2 Plasma and CSF OCN Levels in Different Diagnostic Groups

**FIGURE 1:**
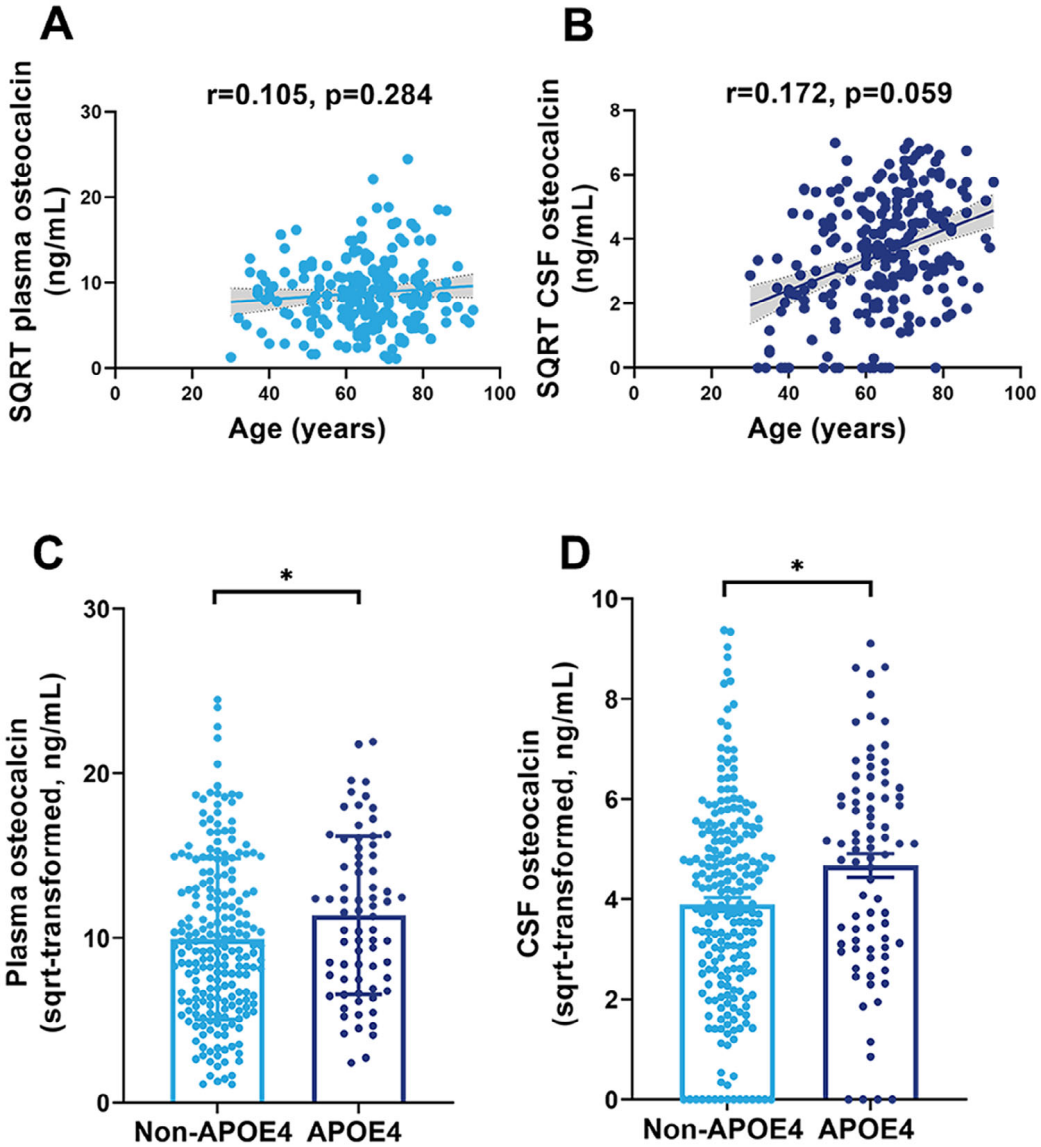
The associations of plasma and CSF osteocalcin levels with age and APOE ε4 status. (A and B) Partial correlations of plasma OCN (A, *n* = 206) and CSF OCN (B, *n* = 238) levels with age in the cognitively unimpaired group with adjustment for sex, APOE ε4 genotype, and education level. (C and D) Comparisons of plasma and CSF OCN levels in the APOE *ε*4 and non‐APOE *ε*4 groups with adjustment for age, sex, APOE genotype, and education level. *p* Values are indicated with asterisks: *p < 0.05. APOE, apolipoprotein E; CSF, cerebrospinal fluid; OCN, osteocalcin; SQRT, square root.

**FIGURE 2:**
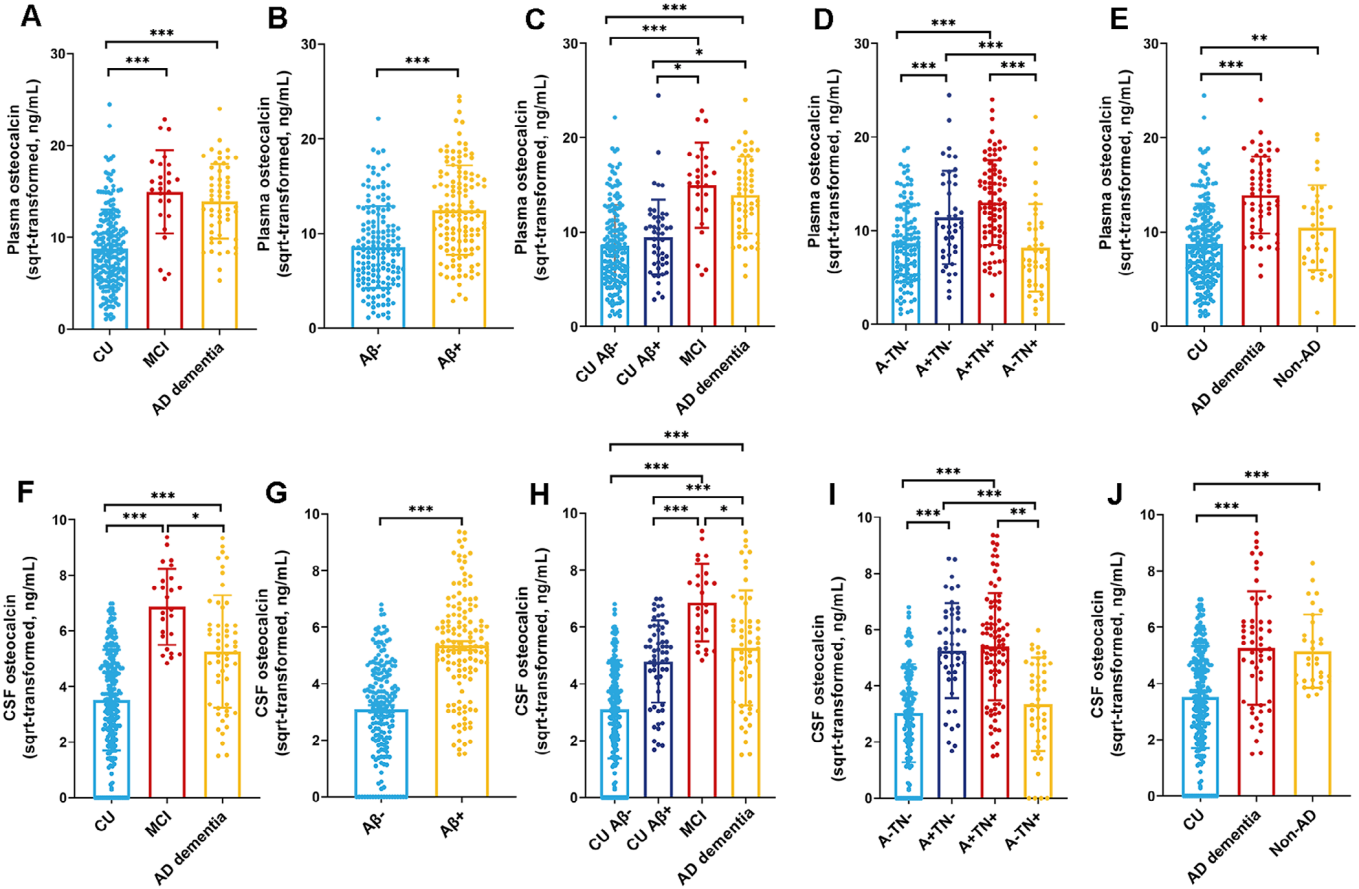
Comparisons of plasma and CSF osteocalcin levels in different groups. (A) Plasma OCN levels in CU (*n* = 206), MCI (*n* = 26) and AD dementia (*n* = 54) groups. (B) Plasma OCN levels in Aβ− (*n* = 158) and Aβ+ (*n* = 128) groups. (C) Plasma OCN levels in CU Aβ− (*n* = 158), CU Aβ+ (*n* = 48), MCI (*n* = 26) and AD dementia (*n* = 54) groups. (D) Comparisons of plasma OCN based on ATN classification (*n* = 286). (E) Plasma OCN levels in CU (*n* = 206), AD dementia (*n* = 54) and non‐AD (*n* = 32) groups. (F) CSF OCN levels in CU (*n* = 238), MCI (*n* = 25), and AD dementia (*n* = 53) groups. (G) CSF OCN levels in Aβ− (*n* = 180) and Aβ+ (*n* = 136) groups. (H) CSF OCN levels in CU Aβ− (*n* = 180), CU Aβ+ (*n* = 58), MCI (*n* = 25), and AD dementia (*n* = 53) groups. (I) Comparisons of CSF OCN based on ATN classification (*n* = 316). (J) CSF OCN levels in CU (*n* = 238), AD dementia (*n* = 53), and non‐AD groups (*n* = 32). Group comparisons were conducted using a one‐way analysis of covariance (ANCOVA) adjusted for age, sex, APOE ε4 genotype, and education level, followed by post hoc multiple comparisons with Bonferroni correction. *p* Values are indicated with asterisks: *p < 0.05, **p < 0.01, ***p < 0.001. AD, Alzheimer's disease; APOE, apolipoprotein E; CSF, cerebrospinal fluid; CU, cognitively unimpaired; CU−, Aβ‐negative cognitively unimpaired; CU+, Aβ‐positive cognitively unimpaired; Aβ−, Aβ‐negative subjects; Aβ+, Aβ‐positive subjects; A−TN−, negative Aβ, p‐tau, and t‐tau; A+TN−, Aβ‐positive while p‐tau and t‐tau‐negative; A+TN+, Aβ‐positive, p‐tau or t‐tau‐positive; A−TN+, Aβ‐negative, p‐tau or t‐tau‐positive; OCN, osteocalcin; SQRT, square root.

We first examined whether plasma and CSF OCN levels were associated with age and APOE ε4 genotype. As shown in Figure 1A,B, age was not significantly correlated with plasma OCN levels (*r* = 0.105, *p* = 0.284) or CSF OCN levels (*r* = 0.172, *p* = 0.059) in CU groups. However, the APOE *ε*4 carriers had higher plasma and CSF OCN levels compared with non‐APOE *ε*4 carriers (Figure 1C,D). In Figure 2, We then compared plasma and CSF OCN levels across diagnostic groups using analysis of covariance (ANCOVA), adjusting for age, sex, APOE ε4 genotype, and education level. In the clinical diagnostic framework, OCN levels were significantly elevated in both plasma and CSF in the MCI and AD dementia group compared with the CU group (Figure 2A,F). Additionally, the MCI group also exhibited higher CSF OCN levels compared with the AD dementia group (Figure 2F). In the ATN biological framework, plasma and CSF OCN levels were significantly higher in Aβ‐positive (Aβ+) participants than in Aβ‐negative (Aβ−) participants (Figure 2B,G). The MCI and AD dementia group also exhibited elevated CSF OCN levels compared with the CU Aβ+ group (Figure 2C,H). Among all participants, plasma and CSF OCN levels were significantly higher in the A+TN+ and A+TN− groups compared with the A−TN− and A−TN+ groups (Figure 2D,I).

Then we included 32 patients with non‐AD neurodegenerative diseases to measured their OCN levels. These participants showed no significant differences in age, sex, or education compared with the CU or AD dementia groups (Table S1). However, the non‐AD group had a lower proportion of APOE ε4 carriers compared with AD dementia groups. As expected, the MMSE scores were reduced in both the AD and non‐AD groups compared with the CU group. Similarly, the CDR score also exhibited elevated in both AD and non‐AD groups. Notably, the non‐AD group also showed higher plasma and CSF OCN levels compared with the CU groups (Figure 2E,J).

### 2.3 Correlations of OCN Levels With Core AD Biomarkers

**FIGURE 3:**
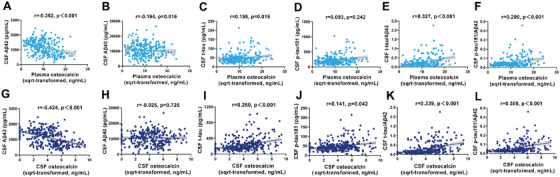
Correlations of plasma and CSF osteocalcin levels with Alzheimer's disease core biomarkers. (A–F) Correlations of plasma OCN with CSF Aβ40, Aβ42, t‐tau, p‐tau181, t‐tau/Aβ42, and p‐tau181/Aβ42 were shown in scatter plots. *n* = 286. (G–L) Correlations of CSF OCN with CSF Aβ40, Aβ42, t‐tau, p‐tau181, t‐tau/Aβ42, and p‐tau181/Aβ42 were shown in scatter plots. *n* = 316. Partial correlation analyses adjusted age, sex, apolipoprotein E genotype, and education level. The shaded areas represent the 95% confidence intervals. CSF, cerebrospinal fluid; OCN, osteocalcin; p‐tau181, phosphorylated tau 181; SQRT, square root; t‐tau, total‐tau.

We analyzed the associations between OCN levels and core AD biomarkers using partial correlation analyses, adjusting for age, sex, APOE ε4 genotype, and education level. As shown in Figure 3A–F, plasma OCN had negative correlations with CSF Aβ42 levels (*r* = −0.362, *p* < 0.001) and Aβ40 levels (*r* = −0.194, *p* = 0.016) and positively correlations with t‐tau (*r* = 0.195, *p* = 0.015), t‐tau/Aβ42 (*r* = 0.327, *p* < 0.001) and p‐tau181/Aβ42 (*r* = 0.290, *p* < 0.001). Similarly, CSF OCN levels were negatively correlated with CSF Aβ42 levels (*r* = −0.424, *p* < 0.001) and positively correlated with t‐tau (*r* = 0.250, *p* < 0.001), p‐tau181 (*r* = 0.141, *p* = 0.042), t‐tau/Aβ42 (*r* = 0.339, *p* < 0.001), and p‐tau181/Aβ42 (*r* = 0.300, *p* < 0.001) (Figure 3G–L).

### 2.4 Correlations of OCN Levels With Cognitive Functions

To further explore the relationship between OCN levels and cognitive functions, we conducted correlation analyses between plasma and CSF OCN levels and MMSE and CDR scores. As shown in Table 2, MMSE scores were negatively correlated with OCN levels in both plasma (*r* = −0.465, *p* < 0.001) and CSF (*r* = −0.454, *p* < 0.001). The CDR scores were positively correlated with OCN levels in plasma (*r* = −0.490, *p* < 0.001) and CSF (*r* = 0.435, *p* < 0.001). The correlations between OCN levels and cognitive function remained significant after adjusting for age, sex, APOE ε4 genotype, and education level.

**TABLE 2:** Associations of plasma and CSF osteocalcin with cognitive functions.

	MMSE scores				CDR scores			
	Unadjusted^a^		Adjusted^b^		Unadjusted^a^		Adjusted^b^	
	*r*	*p* Values	*r*	*p* Values	*r*	*p* Values	*r*	*p* Values
Plasma osteocalcin	−0.465	<0.001	−0.345	<0.001	0.490	<0.001	0.346	<0.001
CSF osteocalcin	−0.454	<0.001	−0.365	<0.001	0.435	<0.001	0.345	<0.001

Abbreviations: CDR, clinical dementia rating; CSF, cerebrospinal fluid; MMSE, Mini‐Mental State Examination.

^a^ Spearman correlation analyses were used to examine the correlations of plasma and CSF osteocalcin with MMSE and CDR scores.

^b^ Partial correlation analyses were adjusted for age, sex, apolipoprotein ε4 genotype, and education level.

### 2.5 CSF OCN Moderated the Relationship Between Aβ Pathology and Tau Pathology

**FIGURE 4:**
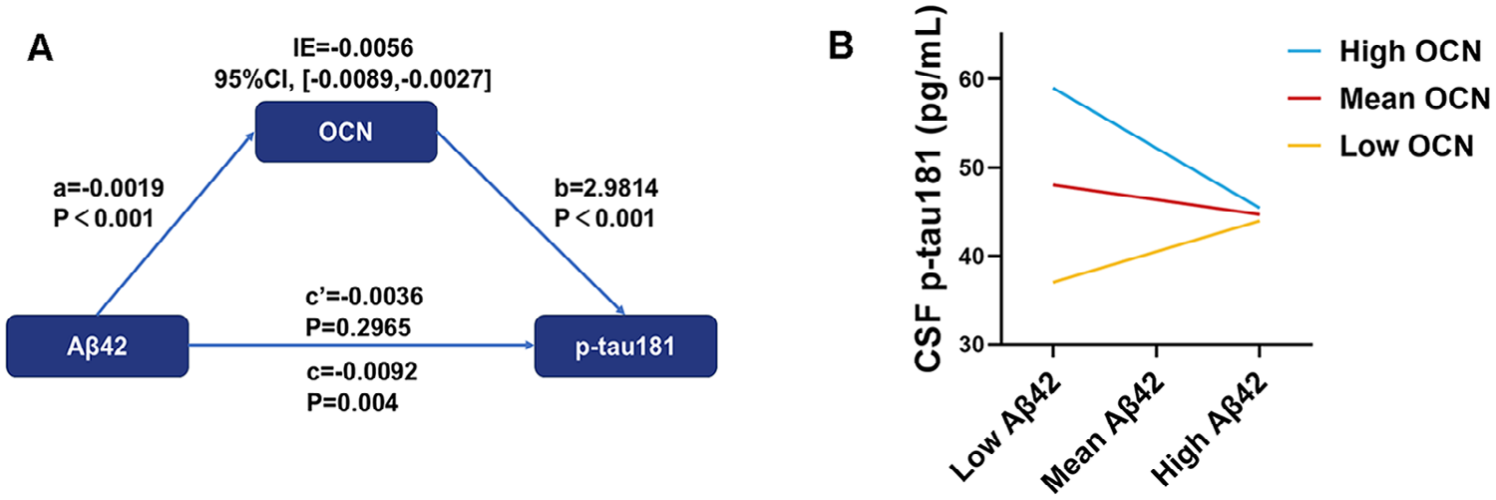
Mediation and moderation effects of CSF osteocalcin on Aβ‐mediated tau hyperphosphorylation. (A) The mediation effect of CSF OCN in mediating the CSF Aβ42 on p‐tau181. (B) The moderation effect of CSF OCN in moderating the CSF Aβ42 on p‐tau181. Low, mean, and high CSF OCN and Aβ42 represent values of mean‐1SD, mean, and mean+1SD, respectively. CI: confidence intervals; CSF, cerebrospinal fluid; OCN, osteocalcin; IE, indirect effect; p‐tau181, phosphorylated tau 181; SD, standard deviation.

We further investigated the mediation effect of OCN in the relationship between Aβ pathology and tau pathology. Our analysis revealed that CSF Aβ42 indirectly influenced CSF p‐tau181 through OCN significantly (Figure 4A). Then we conducted moderation analyses to assess the role of OCN in the interaction between Aβ and tau pathologies. CSF OCN levels significantly enhanced the effect of CSF Aβ42 on CSF p‐tau181 (Figure 4B; Δ*R* ^2^ = 0.0256, *p* = 0.0033).

### 2.6 Diagnostic Performance of OCN in Different Diagnostic Groups

**FIGURE 5:**
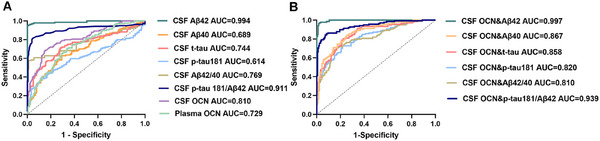
Receiver operating characteristic analysis to discriminate diagnostic groups. (A) AUC of CSF Aβ40, Aβ42, t‐tau, p‐tau181, t‐tau/Aβ42, p‐tau181/Aβ42, OCN, and plasma OCN in the ROC curves when distinguishing Aβ− and Aβ+ participants. (B) AUC of CSF Aβ40, Aβ42, t‐tau, p‐tau181, t‐tau/Aβ42, p‐tau181/Aβ42 combined with CSF OCN in the ROC curves when distinguishing Aβ− and Aβ+ participants. Aβ−, Aβ‐negative subjects; Aβ+, Aβ‐positive subjects; AUC, area under curve; CSF, cerebrospinal fluid; OCN, osteocalcin; p‐tau181, phosphorylated tau 181; t‐tau, total‐tau.

Receiver operating characteristic (ROC) curve analysis was conducted to evaluate the diagnostic potential of plasma and CSF OCN (Figure 5). Results showed that OCN could distinguish between Aβ+ and Aβ− participants, with an area under the curve (AUC) of 0.810 for CSF OCN and 0.729 for plasma OCN (*p* < 0.001) (Figure 5A). Furthermore, we analyzed whether combing CSF OCN with core AD biomarkers could enhance the diagnostic accuracy. As shown in Figure 5B, the addition of CSF OCN significantly improved the AUCs for t‐tau, p‐tau181, and the p‐tau181/Aβ42 compared with using these biomarkers alone. These results suggest that OCN has potential diagnostic value for identifying Aβ+ individuals, either as a standalone biomarker or in combination with core AD biomarkers.

## 3 Discussion

In this study, we identified elevated levels of plasma and CSF OCN in patients with preclinical AD, MCI, and AD dementia. Both plasma and CSF OCN levels were significantly associated with brain Aβ deposition, tau hyperphosphorylation, neurodegeneration, and cognitive decline. Additionally, CSF OCN mediated the relationship between Aβ pathology and tau pathology. To the best of our knowledge, this is the first study to demonstrate that OCN changes occur early in the AD continuum and are strongly linked to brain AD pathologies.

Recent research has identified OCN as a bone‐derived protein that regulates various extra‐skeletal organs, including the brain [8, 16]. The relationship between OCN and cognitive function has become a growing area of interest. Animal studies have demonstrated that OCN could cross the BBB and influence cognitive function [11, 12, 20], but its role in the AD remains unclear. Mendelian randomization studies have suggested that OCN may provide protective effect against AD [21, 22]. A clinical study reported an association between lower OCN levels and poorer cognitive performance [23]. However, this study focused on individuals with obesity, a population distinct from ours, which included biomarker‐diagnosed AD patients. The cognitive assessments used in these studies also differed. Previous small‐scale studies found that blood OCN levels were elevated in patients with AD dementia and negatively correlated with cognitive function, findings consistent with our results [24, 25]. Overall, we speculate that the heterogeneity of OCN levels in different studies may stem from differences in the study populations. Importantly, whether CSF OCN levels are altered in AD and how they relate to core AD biomarkers has not been explored until now. In this study, we analyzed the plasma and CSF OCN levels across different stages of AD and observed that CSF OCN levels were already elevated in the preclinical stages of AD. Furthermore, OCN levels were significantly correlated with core AD biomarkers. We also found that OCN levels were elevated in patients with non‐AD neurodegenerative diseases, such as frontotemporal dementia (FTD) and multiple system atrophy (MSA), suggesting that OCN changes are not specific to AD.

The bone–brain communication axis has recently gained significant research attention [10, 16, 26], with the bone‐derived OCN emerging as a key signal mediator. OCN has been shown to bind to neurons in the brainstem, midbrain, and hippocampus, where it enhances the synthesis of monoamine neurotransmitters and inhibits gamma‐aminobutyric acid production [11]. Exogenous OCN improved hippocampal‐dependent memory and reduced anxiety‐like behaviors in aged mice by binding to G protein‐coupled receptor (GPR) 158, expressed in neurons of the hippocampal CA3 region [12]. Additionally, OCN could regulate the oligodendrocyte differentiation and myelination through interactions with GPR 37 [13]. A recent study also demonstrated that exogenous OCN administration reduced brain Aβ burden and cognitive decline in amyloid precursor protein (APP)/PS1 mice [27]. In this study, we found CSF OCN levels were positively correlated with brain Aβ accumulation and tau levels in humans. Therefore, we hypothesize that the increase in bone‐derived OCN during AD may represent a neuroprotective mechanism of bone–brain axis, potentially influencing neurotransmitter levels, promoting myelination, and mitigating AD pathology. In metabolic disorders such as obesity and impaired glucose regulation, it has been observed that the body compensates by secreting bone‐derived hormones, often compromising bone health [28, 29]. On the other hand, OCN abnormalities may also be the consequence of AD. A series of studies have shown that APP and Aβ can regulate osteoclast differentiation and inhibit osteoblast function [30, 31]. Another study found that in a tau‐based model of AD mouse, reduced bone mineral density was observed before significant tauopathy in the hippocampus and olfactory regions, providing evidence of early bone loss in AD [32]. We propose that elevated OCN levels in AD may be a response to disease‐related bone changes, with pathological processes stimulating osteoblasts to secrete more OCN, potentially leading to bone loss. However, it remains unclear whether there is a direct relationship between Aβ and OCN, warranting further investigation.

Osteoporosis is a skeletal disease characterized by low bone mineral density and the deterioration of bone microarchitecture [33]. Both AD and osteoporosis are prevalent chronic degenerative conditions in the elderly, and their potential connection has garnered increasing attention [34]. It has been suggested that osteoporosis could increase the risk of AD [17–19, 35], however the underlying mechanism remains poorly understood. OCN, a key marker of bone metabolism, is known to be elevated in osteoporosis [9, 24, 36]. This aligns with our hypothesis that elevated OCN levels in AD mat be linked to the higher prevalence of osteoporosis observed in AD patients. Our findings demonstrated that AD patients with worse cognitive function had higher OCN levels, which were associated with core AD biomarkers, suggesting that OCN may be an essential molecule connecting osteoporosis and AD. It indicated that the measurement of OCN may provide an alternative approach for screening AD patients at the early stage. Prospective clinical studies are necessary to investigate the roles of OCN in predicting AD onset and progression in the future.

This study's primary strength lies in its large sample size and the use of clinical and CSF biomarker data as diagnostic criteria. This enables us to examine AD patients in the preclinical stages and include a broader sample of individuals with underlying AD pathology. However, some limitations should be acknowledged. First, as a cross‐sectional study, we cannot establish a causal relationship between OCN and AD. Longitudinal cohort studies are necessary to determine the trajectory of OCN levels throughout the progression of AD or cognitive decline, which could provide deeper insights into OCN's role in AD. Second, bone mineral density tests were not performed in our study. While we excluded patients with osteoporosis based on the use of osteoporosis medications or clinical diagnoses, it remains possible that a small number of osteoporosis patients may have been overlooked. Future studies with more comprehensive designs, including bone density assessments, are needed to validate our findings and minimize the potential impact of confounding factors.

In conclusion, this study provides clinical evidence for the relationship between OCN and AD, indicating that OCN may be associated with brain Aβ deposition, tau hyperphosphorylation and neurodegeneration. While these findings are promising, they require validation in larger, multicenter, and prospective studies. Additionally, the underlying mechanism of OCN in AD is definitely worthy of further investigation in animal models.

## 4 Materials and Methods

### 4.1 Study Participants

Participants in the CADS were recruited through advertising and clinical practice at Daping Hospital, Chongqing, China [37, 38]. The clinical assessment and diagnosis of AD were performed in accordance with the protocol we used previously [38]. Cognitive and functional statuses were evaluated using a neuropsychological battery including the MMSE and CDR scores, and so on, and participants suspected of AD were further underwent PiB‐PET or AV45‐PET imaging to assess brain Aβ deposition. Ethics approval was obtained from the Institutional Review Board of Daping Hospital (No. 2023–106). All participants or their proxies provided informed consent.

In the CADS cohort, the cut‐off values of CSF core AD biomarkers were Aβ42 ≤ 933 pg/mL (A+), p‐tau181>48.7 pg/mL (T+), and t‐tau >313 pg/mL (N+) [39]. According to the ATN framework, participants could be assigned to different groups as follows: A−T−N−, negative Aβ, p‐tau181, and t‐tau; A+, Aβ‐positive; A+TN+, Aβ‐positive, p‐tau181, or t‐tau‐positive; A−TN+, Aβ‐negative, p‐tau181, or t‐tau‐positive [40, 41]. Participants with A−TN+ are classified as having SNAP. Finally, we included 54 Aβ‐PET‐positive AD dementia patients, 26 Aβ‐PET‐positive MCI patients, 238 CU participants (including 136 A−T−N−, 58 A+, and 44 A−TN+), and 32 non‐AD neurodegenerative patients with FTD or MSA. Of the non‐AD cohort, 15 patients with FTD or MSA were recruited from the Second Affiliated Hospital of Zhejiang University. The diagnostic criteria of AD, MCI and preclinical AD were defined in compliance with the National Institute on Aging–Alzheimer's Association workgroups diagnostic criteria [41, 42, 43]. The FTD and MSA patients were diagnosed according to the consensus clinical diagnostic criteria of FTD [44, 45], and the second consensus statement on the diagnosis of MSA [46], respectively. The sample size for this study was determined using the PASS software to ensure an *α* of 0.05, *β* of 0.2, a permissible error of 0.6 (calculated from the difference between AD and healthy controls) [25], and a standard deviation (SD) of 1.88 as reported in a previous study [25]. The calculated sample size was 82, ensuring that our study was adequately powered to detect the associations.

### 4.2 APOE Genotypes and Aβ‐PET Acquisition

*APOE* genotypes (rs429358 and rs7412) were determined using the polymerase chain reaction‐restriction fragment length polymorphism method [47]. Brain Aβ‐PET imaging was conducted according to the protocol described previously [48].

### 4.3 AD Core Biomarkers and OCN Measurements

Blood samples from all participants were collected after an overnight fast. CSF and plasma were centrifuged at 2000×*g* for 10 min within 2 h to eliminate cells and other insoluble material, and snap frozen at −80°C until analysis. The thaw/freezing cycle was limited to no more than two times. In accordance with the global measurement standardization from the Alzheimer's Association Global Biomarkers Consortium [49], CSF Aβ42, Aβ40, t‐tau, and p‐tau181 levels in the CADS cohort were measured at the laboratory of the Neurology department in Daping Hospital, which is a member of the Alzheimer's Association Quality Control Program. Briefly, CSF Aβ42, Aβ40, t‐tau, and p‐tau181 were measured by commercially available enzyme‐linked immunosorbent assay (ELISA) kits (Innotest, Fujirebio Europe, Gent, Belgium) or chemiluminescent enzyme immunoassays using the Lumipulse G 1200 automated platform (Fujirebio Europe). Plasma and CSF OCN levels were measured at the laboratory of the Neurology department in Daping Hospital using human OCN ELISA kits (RayBiotech Life, Peachtree Corners, Georgia, USA). All assays were performed by professional experimenters who were blinded to the clinical information and strictly followed the manufacturer's protocol procedures.

### 4.4 Statistical Analysis

Continuous variables are presented as the mean ± SD. The Kolmogorov–Smirnov test was used to assess whether continuous variables followed a normal distribution. Demographic variables (age, education, MMSE, CDR, Aβ40, Aβ42, t‐tau, p‐tau181) were compared across groups via one‐way analysis of variance (ANOVA). Categorical variables (sex, APOE, diabetes, hypertension) were expressed as a proportion (%) and compared across groups via chi‐square test. To normalize their distributions for subsequent analyses, plasma and CSF OCN levels were transformed using the square root (SQRT) method. Differences among groups were determined by an independent‐samples *t*‐test or one‐way ANOVA, or ANCOVA with adjustment for age, sex, APOE ε4 genotype, and education level and Bonferroni correction for multiple comparisons. Partial correlation analyses were performed to assess the correlations between plasma and CSF OCN with AD core biomarkers and MMSE and CDR scores with adjustment for age, sex, APOE ε4, and education level. Moderation and mediation analyses were conducted to examine whether the relationships between CSF Aβ42 and p‐tau181 could be influenced by CSF OCN. These analyses were performed by the PROCESS macro in SPSS using 5000 bootstrap samples. The diagnostic value of CSF OCN was assessed by estimation of the AUC from ROC curves. All tests were two‐sided, and *p *< 0.05 was considered statistically significant. All statistical analyses were performed using IBM SPSS Statistics version 27 or GraphPad Prism 9.0.

## Author Contributions

Yan‐Jiang Wang and Xian‐Le Bu conceptualized and designed the study. Yan‐Jiang Wang, Xian‐Le Bu, Zhuo‐Ting Liu, Jia‐Yan Xin, and Mei Huang contributed to the data interpretation and manuscript drafting and critical revision. Yu‐Di Bai, Jin Zhou, Yun‐Yu Bao, Jiang‐Hui Li, Zhi‐Hao Liu, Gui‐Hua Zeng, An‐Yu Shi, Dong‐Wan Chen, Yu‐Jie Lai, Yang Chen, Fan Zeng, Jun Wang, Qing‐Qing Tao, and Zhi‐Ying Wu contributed to the data collection, data analysis, data interpretation, and revision of the manuscript. All authors have read and approved the final manuscript.

## Ethics Statement

Ethics approval was obtained from the Institutional Review Board of Daping Hospital (No. 2023–106). All participants or their proxies provided informed consent.

## Conflicts of Interest

The authors declare no conflicts of interest.

## Supporting information



Supporting Information

## Data Availability

Data are available upon reasonable request from the authors.
