# Supplementary material for: Associations of Plasma and CSF Osteocalcin Levels With CSF ATN Biomarkers and Cognitive Functions in Alzheimer's Disease
Source: MedComm (2020). 2025 Jun 19;6(7):e70255. doi: 10.1002/mco2.70255 (PMC12179405; doi:10.1002/mco2.70255)
Supplement: Supplementary file 1 — Supporting Information [file MCO2-6-e70255-s001.docx]

**Title:** **Associations of plasma and CSF osteocalcin levels with CSF ATN biomarkers and cognitive functions in Alzheimer’s disease**

**Running title:** **Associations of OCN with AD biomarkers**

Xian-Le Bu^1,2,3,4,5,#,*^, Zhuo-Ting Liu^1,2,5,#^, Jia-Yan Xin^1,2,5,#^, Mei Huang^1,2,5,#^, Yu-Di Bai^1,2,5^, Jin Zhou^1,2,5^, Yun-Yu Bao^1,2,5^, Jiang-Hui Li^1,2,5^, Zhi-Hao Liu^1,2,5^, Gui-Hua Zeng^1,2,5^, An-Yu Shi^1,2,5^, Dong-Wan Chen^1,2,5^, Yu-Jie Lai^1,2,5^, Yang Chen^1,2,5^, Fan Zeng^1,2,3,4,5^, Jun Wang^1,2,3,5^, Qing-Qing Tao^6^, Zhi-Ying Wu^6^, Yan-Jiang Wang^1,2,3,4,5,*^

1. Department of Neurology and Centre for Clinical Neuroscience, Daping Hospital, Third Military Medical University, Chongqing 400042, China

2. Chongqing Key Laboratory of Ageing and Brain Diseases, Chongqing 400042, China

3. Institute of Brain and Intelligence, Third Military Medical University, Chongqing 400038, China

4. State Key Laboratory of Trauma and Chemical Poisoning (Third Military Medical University), Chongqing 400042, China

5. Key Laboratory of Geriatric Cardiovascular and Cerebrovascular Disease Research, Ministry of Education of China, Chongqing 400042, China

6. Department of Medical Genetics and Center for Rare Diseases, and Department of Neurology in Second Affiliated Hospital, and Key Laboratory of Medical Neurobiology of Zhejiang Province, Zhejiang University School of Medicine, Hangzhou, Zhejiang 310009, China

^#^ These authors contributed equally to this work

Corresponding authors: Xian-Le Bu ([buxianle@tmmu.edu.cn](mailto:buxianle@tmmu.edu.cn)),ORCID: 0000-0002-2331-3339; Yan-Jiang Wang ([yanjiang_wang@tmmu.edu.cn](mailto:yanjiang_wang@tmmu.edu.cn)), ORCID：0000-0002-6227-6112

**Table S1. Demographics and clinical data of the cognitively unimpaired participants and patients with AD dementia, and non-AD neurodegenerative diseases.**

| Variable | Cognitively unimpaired (n=238) | AD dementia (n=54) | Non-AD neurodegenerative diseases (n=32) | P  values | η^2^/  Cramer's V |
| --- | --- | --- | --- | --- | --- |
| Age, mean (SD), y | 63.8 (13.4) | 63.4 (9.2) | 63.0 (10.7) | 0.929 | <0.001 |
| Female, n (%) | 80 (33.6) | 32 (59.3) ^a^ | 15 (46.9) | 0.001 | 0.200 |
| Education, mean (SD), y | 10.7 (2.4) | 9.6 (2.6) ^a^ | 9.8 (4.0) | <0.001 | 0.062 |
| APOE ε4 carriers, n (%) | 44 (18.5) | 26 (48.1) ^a^ | 3 (9.4) ^b^ | <0.001 | 0.282 |
| MMSE, mean (SD) | 27.3 (2.3) | 12.3 (5.5) ^a^ | 18.6 (8.5) ^a, b^ | <0.001 | 0.701 |
| CDR, mean (SD) | 0 (0) | 1.6 (0.7) ^a^ | 1.1 (0.7) ^a, b^ | <0.001 | 0.765 |

Categorical variables are presented as numbers and percentages; continuous variables are presented as mean±SD. The p-value in the penultimate column refers to the statistical significance of comparisons between multiple groups determined by one-way analysis of variance and chi-square test. η^2^ and Cramer's V were used to quantify ANOVA and chi-square test effect sizes, respectively. ^a^ p<0.05 compared to cognitively unimpaired, ^b^ p<0.05 compared to AD dementia.

Abbreviations: AD, Alzheimer’s disease; ANOVA, analysis of variance; APOE, apolipoprotein E; CDR, Clinical dementia rating; MMSE, Mini–Mental State Examination; n, number; SD, standard deviation; y, years.
